# Targeted MinION sequencing of transgenes

**DOI:** 10.1038/s41598-020-71614-6

**Published:** 2020-09-15

**Authors:** Anne-Laure Boutigny, Florent Fioriti, Mathieu Rolland

**Affiliations:** Anses, Plant Health Laboratory, Bacteriology Virology GMO Unit, 7 rue Jean Dixméras, 49044 Angers Cedex 01, France

**Keywords:** Plant sciences, Plant genetics, Transgenic plants

## Abstract

The presence of genetically modified organisms (GMO) is commonly assessed using real-time PCR methods targeting the most common transgenic elements found in GMOs. Once the presence of GM material has been established using these screening methods, GMOs are further identified using a battery of real-time PCR methods, each being specific of one GM event and usually targeting the junction of the plant genome and of the transgenic DNA insert. If, using these specific methods, no GMO could be identified, the presence of an unauthorized GMO is suspected. In this context, the aim of this work was to develop a fast and simple method to obtain the sequence of the transgene and of its junction with plant DNA, with the presence of a screening sequence as only prior knowledge. An unauthorized GM petunia, recently found on the French market, was used as template during the development of this new molecular tool. The innovative proposed protocol is based on the circularization of fragmented DNA followed by the amplification of the transgene and of its flanking regions using long-range inverse PCR. Sequencing was performed using the Oxford Nanopore MinION technology and a bioinformatic pipeline was developed.

## Introduction

During the past decade, the presence of genetically modified (GM) plants on the market has considerably increased^1^. According to the European Union (EU) Directive 2001/18/EC on the deliberate release into the environment of genetically modified organisms (GMO) and repealing Council Directive 90/220/EEC^2^, GMOs must be authorized by the national competent authority before being placed on the market or imported into the EU. In this context, national reference laboratories are in charge of territory surveillance and control the presence of GMOs in crops, food and feed. The presence of GMOs is currently assessed by reference laboratories using real-time PCR methods screening transgenic elements commonly found in GMOs, such as the Cauliflower mosaic virus (CaMV) 35S promoter (p35S) and the nopaline synthase terminator from *Agrobacterium tumefaciens* (tNOS)^3^. When a screening method detects GM material, a battery of real-time PCR methods targeting the junction sequence between the plant genome and the transgenic DNA insert is used to identify the present GMO(s). This junction is unique for each GM event^4^. For GM plants authorized in the EU, event-specific detection methods and certified reference material are both provided by GMO developers. When a screening method detects GM material, but no GM event specific method allows the identification of the present event, the presence of an unauthorized GMO is suspected. In the context of unauthorized GMOs, a rapid molecular tool to sequence the transgene and its flanking region would be of great interest to rapidly design a new real-time PCR event-specific detection method.

In 2017, unauthorized transgenic petunia plants were detected on the European market^5^. These plants contained the A1 gene from *Zea mays* L. encoding the dihydroflavonol reductase which was first introduced into a petunia to study the flavonoid metabolic pathway^6^. These petunias also had the particularity of producing orange flowers not previously seen in the genus^6^. Such plants have never been through the authorization process required in the EU and should not have been commercialized. In this context, numerous GM petunia plants had to be withdrawn and destroyed from the European market in 2017. The construct inserted in the genome of the detected GM petunia contained the A1 gene but also the p35S promoter. The GM petunia could therefore be detected using screening methods targeting these sequences but could not be identified using event-specific detection method.

With the development of high throughput next generation sequencing (NGS) technology, whole genome sequencing (WGS) strategies have been used to characterize transgenes and insertion sites by comparing the sequence of the reads obtained from the GMO or GM product with a reference genome of the same species^7^ or with databases of known transgenes^8^. WGS has been successfully applied to describe transgenes in GM rice, soybean and flax^9–13^. However, the transgene represents a very small fraction of the whole genome, for example 4,000 bp in a 1.4 Gbp considering GM petunia (0.0002%)^6,14^ and a high depth of coverage is required to allow the detection of a small amount of a given GMO^15,16^.

Considering the large genome of most plants, targeted NGS strategy provides a cheaper and faster alternative to WGS by sequencing only sequences of interest. Common targeting and enrichment strategies include PCR amplification and hybridization-based capture. These strategies require a minimum of prior knowledge to target the sequences of interest. PCR-based DNA walking methods anchored on the detected transgenic elements, for instance p35S, tNOS, t35S pCAMBIA, or VIP3A elements, have been applied to GMOs including GMOs at trace levels, GMO mixtures and processed food matrices. The resulting amplicons were sequenced using NGS technology and the transgene sequence and its flanking regions could be characterized^17–25^. Although effective, this technique is difficult to implement and remains time consuming. Recently, an approach combining NGS with a strategy of enrichment for the regions of interest was developed for the detection and characterization of GM events^26^. Regions of interest were captured using probes targeting 40 structural elements commonly used in genetically modified plants. After enrichment, the DNA libraries were sequenced on an Illumina MiSeq system allowing the partial or complete reconstruction of the GM sequence inserted into the plant genome^26^. However, the cost for DNA enrichment and sequencing and the time required to obtain the results, especially if the sequencing is subcontracted, are high. Therefore, the use of affordable instruments like the Oxford Nanopore MinION sequencer should be considered. In addition, long-read sequencing instruments, such as the Pacific Biosciences or the MinION sequencers, could provide reads covering the whole transgene. This approach would facilitate the assembly of the reads.

**Figure 1:**
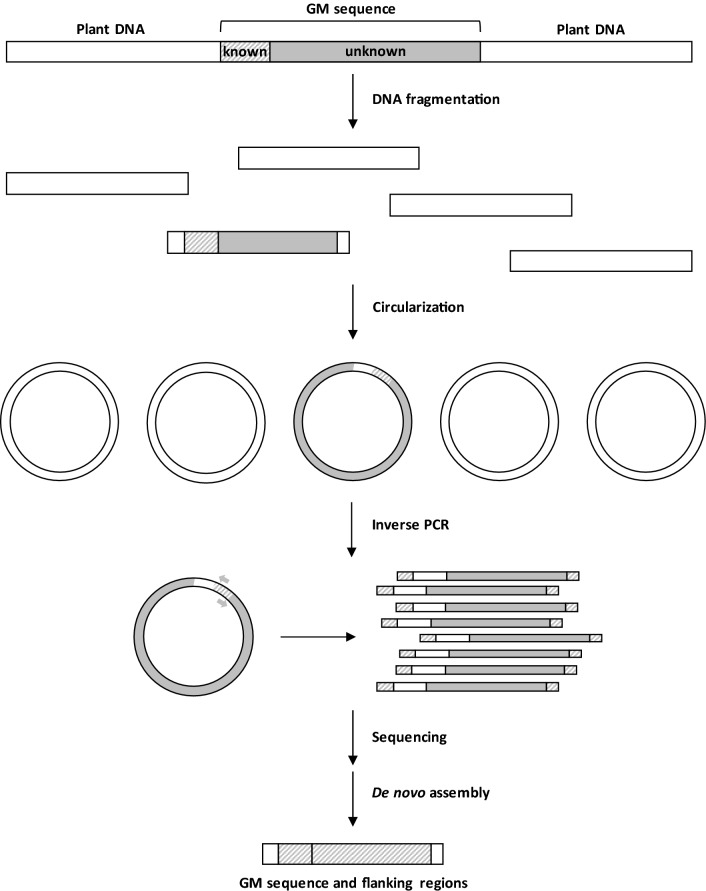
Overview of the protocol developed in this study. DNA was sheared into 6 kb fragments, end-repaired and circularized. An inverse long-range PCR, using p35S primers orientated in the reverse direction, specifically amplified circularized DNA molecules that contained the p35S promoter. PCR products containing the transgene or a portion of the transgene were sequenced using the Oxford Nanopore sequencer. The bioinformatic pipeline allowed to recover the sequence of the transgene and its flanking regions.

In the unauthorized GMO context, our aim was to develop a rapid and simple method to sequence the transgene and its flanking regions with, as prior knowledge, the presence of a detected screening sequence. The method was developed and assessed on available GM petunia plants, for which part of the transgene sequence was available on NCBI and of which the 5′ flanking region had recently been described^17^. In 1988, Ochman et al. described a protocol of inverse PCR allowing the amplification of regions of unknown sequence flanking a specified segment of DNA^27^. The method uses PCR amplification, but the primers are orientated in the reverse direction and the template for the reverse primers is a restriction fragment ligated upon itself to form a circle^27^. In the present work, we developed an innovative protocol (Fig. 1) to amplify the transgene and its flanking regions based on inverse long-range PCR targeting p35S on circularized molecules of approximately 6 kb. Sequences of interest were further sequenced using Oxford Nanopore long read sequencing and a bioinformatic pipeline was developed.

## Results and discussion

In order to amplify and sequence the petunia transgene and its flanking regions, a new protocol was optimized in this study (Fig. 1). First, high-quality and high-molecular-weight genomic DNA was extracted using a commercial kit based on magnetic purification. Then, DNA was sheared into 6 kb fragments, end-repaired and circularized. An exonuclease treatment allowed digestion of non circularized fragments. An inverse long-range PCR, using p35S primers orientated in the reverse direction, specifically amplified circularized DNA molecules containing the p35S promoter. PCR products (all containing a portion of the transgene) were sequenced using the Oxford Nanopore sequencer.

**Figure 2:**
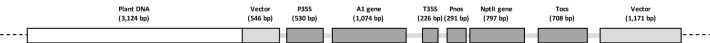
Petunia GM sequence and its flanking regions obtained in this study. *P35S* 35S promotor, *T35S* 35S terminator, *Pnos* nos promotor, *Tocs* ocs terminator.

A total of 131,031 reads were produced and basecalled and of these, 98,111 (74.9%) passed the quality control. The mean read quality was 12.2. The initial shearing into 6 kb fragments was approximate. The visualization of the PCR amplicons on an agarose gel showed that the size of the amplicons was comparable to the size of the sheared DNA (data not shown). It cannot be excluded that shearing happened during the library preparation step as the mean read length was 1,879 bp. Longer reads were also observed, the longest one being 9,538 bp. The final sequence obtained corresponded to the assembly of 2 smaller contigs, it was 9,287 bp long and covered 3,124 bp of plant DNA and 6,168 bp of transgene (Fig. 2). The sequencing depth went from 1,005 to 46,641, with an average depth of 15,866. Sequencing depth was higher for positions located close to the primers used for amplification. After the final polishing step, our sequence presented a 99.9% similarity with the available sequence (MF521566.1) on the 3,304 bp they shared. The obtained sequence was annotated using alignment on NCBI database (Fig. 2) and deposited in GenBank under accession number MT178397. The 5′ end of the sequence corresponded to plant DNA and the junction between the plant DNA and the transgene was identified. Cloning vector sequences were identified at the extremities of the transgene and several genetic elements could be annotated: the 35S promotor, the A1 gene, the 35S terminator, the nos promotor, the NPTII gene, the ocs terminator. These elements and their arrangements correspond to the plasmid p35S A1 which was used for the transformation of Petunia in the original work of Meyer et al. (1987). This suggests the commercial use of the orange flowers GM petunia primarily developed for a research purpose. The transgene sequence obtained in this work (6,168 bp) was longer compared to the 2 sequences available in the NCBI database (3,304 bp for MF521566.1 and 3,627 bp for KY964325.1). Although the same genetic elements could be identified, our sequence is the only including a junction between the plant genome and the transgene and the sequence of this junction is needed to develop an event specific real time PCR test. At the 3′ end, our sequence does not cover the whole cloning vector, the initial plasmid P35 A1 being approximately 8 kb^6^. In order to identify the junction between the transgene and plant DNA at the 3′ end, initial DNA could be sheared into longer fragments. Another strategy would be to apply the same approach using a PCR targeting a screening element present at the 3′ end of the transgene. This method is transferable to any other screening element by simply performing the inverse PCR with primers corresponding to the reverse complement of the initial primers used for the detection of the screening element by real-time PCR. It is applicable to any genetically modified organism including microorganisms or animals. As presented here, it can be used to sequence an unknown transgene in a diagnostic context, but it is also applicable to identify the site of insertion of a known construct during the development of a new GMO.

As shown by the example of the GM petunia, there is a risk of misuse of genetically modified biological material developed for research purposes. Furthermore, the risk of finding such material on the market is increased when the transgene provides a trait of commercial interest^28^.

Based on the data currently available, GM petunias represent negligible risks to human health and safety and to the environment. Indeed, these plants are not persistent, they are not consumed and the risk of a transfer of the transgene to the environment is extremely weak^29^. Nevertheless, in 2017, the massive destruction of GM petunias in Europe caused economic losses. It cannot be excluded that research GM plants representing sanitary or environmental risks could be found on the market if used involuntarily in breeding programs. In addition, unauthorized GMOs could reach the market in imports due to the increasing cultivated area and number of GM cultivated worldwide, asynchronous and asymmetric authorizations outside and within the EU^30^. At the first detection of an unauthorized GMO, this method will allow the sequencing of the transgene and of the insertion site. Based on these newly acquired data, it will be possible to rapidly develop an event-specific detection method, which will allow the specific quantification of sequenced GM material and its differentiation from other potential authorized or unauthorized events. Considering that this method is not meant to be used for routine testing but only for the sequencing of new unknown events, its cost is very reasonable.

Recently, an enrichment strategy using targeted cleavage with Cas9 for ligating nanopore sequencing adaptors at specific locations has been developed and termed ‘nanopore Cas9 Targeted-Sequencing’^31^. This new method was demonstrated to allow the evaluation of targeted genomic sites simultaneously for DNA methylation patterns, structural variations or small mutations^31^. In the context of the present manuscript, this strategy could be applied to specifically cut DNA and ligate sequencing adaptors at a known location of a transgene, in order to sequence the transgene and the bordering host genome. However, all of these targeting methods (PCR amplification, hybridization-based capture, and CRISPR/Cas-mediated enrichment) have some limits as they can only be applied to GM material after a positive screening result has been obtained by targeting a known sequence (e.g. a promotor or terminator classically used to create GMO). The targeted sequencing protocol developed in our study could be applied in several other applications like assessing medically relevant genes, analyzing gene organization, locating viral integration or sequencing the insertion sites of any known mobile genetic element.

Processing the basecalled data using Shasta^32^, a de novo long-read assembler, allowed a fast and accurate assembly. However, on the obtained contigs, the extremity corresponding to the known sequence was missing. To be able to reconstruct the sequence, a second assembly was performed in parallel using SPAdes^33^, an assembler commonly used for Illumina or IonTorrent reads and capable of providing hybrid assemblies using Oxford Nanopore reads. Using fragments of the long reads, this assembler allowed the recovery of the missing sequence, at the junction between the Shasta contigs and the known sequence. This strategy allowed the completion of the full sequence.

With a mean read quality of 12.2, corresponding to an approximate call accuracy of 94%, the Oxford Nanopore MinION technology offers low quality results compared to a platform such as the Ilumina MiSeq which claims to provide 90% of bases with a call accuracy higher than 99.9%^34^. However, in the context of targeted sequencing, this error rate is compensated for by the high depth of coverage. In the present study, a 30 min sequencing run provided an average depth of 15,866 bases. From the raw data available, the use of Shasta and Medaka to assemble and polish the sequence, allowed to obtain an accurate final sequence presenting 99.9% of similarity with the previously available sequence. As expected, the final sequence is therefore suitable to design primers for further use. Despite this low call accuracy, the Oxford Nanopore MinION system offers two major advantages compared to other systems, the ability to generate long reads and the low cost of the equipment. Long read sequencing facilitates the downstream bioinformatics analysis process. The operating cost of the Oxford Nanopore MinION system is not very different from the operating cost of other systems. However, the investment required to acquire other systems is much more important. The low price of the Oxford Nanopore MinION equipment is important as it allows its acquisition by the laboratories, which can then implement the protocol without the need for service providers and complete sequencing of a new GMO in only two days.

## Materials and methods

### Plant materials

GM (African sunset variety) and non GM (mixed varieties) petunia seeds were stored at 4 °C. Plants were maintained in a greenhouse at 25 °C.

### DNA extraction

Each extraction was carried out from 500 mg of freshly harvested petunia leaves. Plant material was ground in liquid nitrogen using a mortar and pestle to obtain a fine homogenous powder. High‐molecular‐weight DNA extraction was performed using the QuickPick SML Plant DNA kit (Bio-Nobile, Pargas, Finland). Ground plant material was homogeneously mixed with 1,500 μL of QuickPick SML Plant DNA lysis buffer and 100 μL of proteinase K solution. The mixture was then incubated 30 min at 65 °C. During this lysis step, QuickPick SML Plant DNA reagents were distributed as follows into 5 strips of 5 tubes:tube 1:10 μL of magnetic particles and 250 μL of binding buffer, tube 2–4:500 μL of wash buffer and tube 5:100 μL of ultra-pure water. After incubation at 65 °C, the lysed plant material was centrifuged for 5 min at 18,000 g. 330 μL of supernatant were gently transferred into each first tube of the 5 strips (binding buffer, magnetic particles). DNA extraction was then performed according to the manufacturer’s instructions using the BioSprint15 workstation (Qiagen, Hilden, Germany). After elution, the content of the last tube of the 5 strips was pooled. RNase A was added at a final concentration of 40 μg/mL and incubated 30 min at 37 °C. The DNA extract was then purified by addition of chloroform (1 volume) and mixed by inversion. After centrifugation at 18,000 g for 20 min, the supernatant was transferred to a new tube. The DNA was further purified by ethanol precipitation. Two volumes of ice-cold 100% ethanol were added to the tube which was then stored for at least 30 min at − 20 °C. After centrifugation at 18,000 g for 10 min at 4 °C, the supernatant was discarded and the resulting pellet was washed with 1 volume of freshly prepared ice-cold 80% ethanol. After centrifugation at 18,000 g for 5 min at 4 °C, the supernatant was discarded and the tube was dried for 10 min at 37 °C. DNA was resuspended in 100 μL ultra-pure water. DNA yields were assessed according to a fluorimetric-based method (Qubit 2.0 Fluorometer; Invitrogen, Carlsbad, CA, USA) using the Qubit dsDNA HS Assay Kit (Invitrogen). DNA quality was assessed by measuring the absorbance of the solution at 230, 260 and 280 nm with a Multiskan GO (Thermo Fisher Scientific, Waltham, MA, USA) spectrophotometer, A260/280 ratio was expected to be between 1.8 and 2 and A260/230 ratio between 2 and 2.2. The integrity of the DNA was evaluated on a 0.5% agarose gel in 0.5X TBE (Tris Borate EDTA).

### Inverse PCR

DNA was sheared into 6 kb fragments using g-TUBE (Covaris, Brighton, England) in an Eppendorf 5,424 centrifuge according to manufacturer’s instructions. Fragmented DNA was end repaired using the NEBNext End Repair Module (New England Biolabs, Evry, France) and purified using AMPure XP beads (Beckman Coulter, Brea, CA, USA) according to manufacturer’s instructions. Circularization of DNA was performed using the Blunt/TA Ligase Master Mix (NEB) by mixing 50 ng of DNA, 1X Blunt/TA ligase and ultra-pure water in a final volume of 50 μL and incubating 1 h at room temperature. DNA sample was purified using AMPure XP beads and eluted in 25 μL of ultra-pure water. Linear DNA was degraded using Exonuclease V (NEB) according to manufacturer’s instructions in a final volume of 30 μL and incubated 30 min at 37 °C and 30 min at 70 °C. The inverse PCR was performed using Q5 hot start Master Mix 1 X (NEB), 500 nM of primer F (35S-F circle 5′-CAATCCACTTGCTTTGAAGACG-3′), 500 nM of primer R (35S-R circle 5′-AATCCCACTATCCTTCGCAAGA-3′), 5 μL of DNA and ultra-pure water in a final volume of 25 μL. The conditions of the PCR were 98 °C for 1 min, followed by 40 cycles at 98 °C for 10 s, 65 °C for 15 s and 72 °C for 4 min 40 s, and a final extension at 72 °C for 2 min.

### Library preparation and sequencing

Libraries were prepared for MinION sequencing (Oxford Nanopore Technologies, Oxford, England) using the 1D amplicon/cDNA by Ligation Sequencing Kit (SQK-LSK109) according to manufacturer’s instructions. The entire library was loaded onto a flowcell (FLO-MIN106D R9) and run for 30 min.

### Sequencing analysis

**Figure 3:**
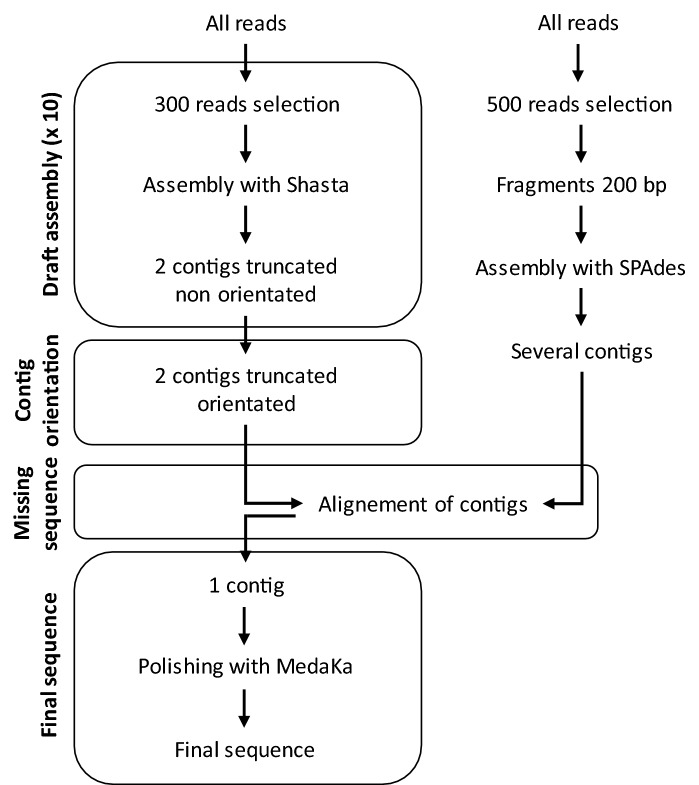
Bioinformatic workflow developed in this study to characterize transgenes.

The bioinformatic pipeline is outlined in Fig. 3. The fast5 files containing raw reads were basecalled using Guppy (v3.0.3; Oxford Nanopore Technologies) in default mode with a quality threshold set at 10. Nanopore summary statistics were obtained using BasicQC (Oxford Nanopore Technologies). The fastq files were converted into a fasta file containing all the reads using an inhouse script. Reads with a length greater than 1,000 bp and containing one of the primers (35S F circle and 35S R circle) were selected with Blast^35^. 300 of these reads were randomly selected to perform a draft assembly using Shasta (v0.1.0). The operation of selecting 300 reads to perform a draft assembly was repeated ten times. The two longest different contigs obtained from the assembly iterations were then selected for further processing. These two contigs corresponded to sequences on either side of the known sequence, however, there relative orientation was unknown. The orientation of the contigs was determined using blast, by searching for the known sequence within the contigs. In parallel, a SPAdes assembly was performed to improve the coverage of the sequence. 500 reads were randomly selected out of the reads of more than 1,000 bp containing one of the primers (35S F circle and 35S R circle). These reads were split into fragments of 200 bp, which were assembled using SPAdes (v3.13.1). Using blast, SPAdes contigs were aligned with those obtained with Shasta. When a match was found and when the SPAdes contig covered a portion of the genome not covered by the Shasta contig, this sequence was used to extend the Shasta contigs. Finally, contigs were polished using MedaKa (v0.8.0; Oxford Nanopore Technologies) to produce the final consensus sequence. The bioinformatic pipeline is freely available for research use at https://github.com/RollandMathieu/Targeted-Minion-sequencing-of-transgenes.
